# Imagery Rescripting: Exploratory Evaluation of a Short Intervention to Reduce Test Anxiety in University Students

**DOI:** 10.3389/fpsyt.2020.00084

**Published:** 2020-02-28

**Authors:** Anna Maier, Caroline Schaitz, Julia Kröner, Bernhard Connemann, Zrinka Sosic-Vasic

**Affiliations:** Department for Psychiatry and Psychotherapy, University Clinic of Ulm, Ulm, Germany

**Keywords:** imagery rescripting, test anxiety, psychotherapy, mental images, imagery, short time intervention

## Abstract

**Background:**

Test anxiety is common in university students. Demanding schedules may contribute to the relatively low utilization of professional counseling, when compared to other anxiety disorders. A possible solution could be a psychotherapeutic short-term intervention. The present exploratory study implemented a short-term psychotherapeutic treatment, consisting of two imagery rescripting (IR) sessions. The efficacy of IR techniques has already been demonstrated in the treatment of various anxiety disorders including test anxiety.

**Methods:**

Nine students suffering from test anxiety (m = 3, f = 6) underwent two weekly applied sessions of IR. Outcome variables were examined one week prior to (t1), immediately after (t2) and three months after (t3) the intervention, using self-evaluation questionnaires on test anxiety (PAF), depressive symptoms (BDI II), life satisfaction (FLZ), general self-efficacy and study-specific self-efficacy (WIRKALL; WIRK_STUD), intrusiveness of mental images (IFES), and change and acceptance (VEV; ZUF; BFTB).

**Results:**

There were no dropouts. According to results from ZUF and BFTB, the participants where highly satisfied with the intervention. PAF scores decreased significantly over time (t1 to t3), whereas WIRKALL scores (t1 to t3) and WIRK_STUD scores (t1 to t3 and t2 to t3) increased significantly. IFES scores decreased immediately after the intervention (t1 compared to t2) and further after the follow-up (t1 to t3). No changes in BDI-II scores were observed.

**Discussion:**

The findings indicate a high acceptance and efficacy of the two-session IR-intervention. Limits are the lack of a control group, and the small sample size. Further evaluation in future controlled studies is needed.

## Introduction

Within our western world, exams are a crucial part of every person’s life (1). Since exams serve a key role within performance evaluation and the resulting decision processes, it is obvious that individuals depend strongly on the results, which are deciding about the individual´s future success (for example job opportunities). Therefore, it is not surprising that the prevalence of test anxiety is on the rise.

In particular, test anxiety is a common phenomenon in students. A German survey by one of the German main public insurances (2, 3), reported that almost a quarter of the questioned students were often stressed, mostly regarding upcoming exams. Females and males are almost equally affected, with women (58%) being slightly more impacted than men (46%). Another representative study found that 42% of undergraduate students had fears related to studying and 17.5% were particularly test anxious (4). This problem is also noticeable in the psychosocial counseling centers of the universities, as can be seen in a German study. Nearly half of the investigated students sought professional help due to test anxiety at the respective counseling center (5). Internationally, the incidence of test anxiety in students varies considerably. Cizek and colleagues (6) summarized prevalence data from various studies ranging from 1% in school-aged children (7) to 41% in third-through sixth grade African-American children (8).

Nevertheless, until today, there are no reliable international studies on the prevalence of test anxiety in students. One major reason might be the inconsistent definition of test anxiety. The disorder is not individually included as a separate diagnosis in either the ICD-10 [(9)] or the DSM-5 [(Diagnostic and Statistical Manual of Disorders (9). Test anxiety is either diagnosed within the specific (isolated) phobias (F40.2) of the situational type, without having its own specific diagnostic criteria; or as a specific form of social anxiety (F40.1). Despite the challenges of a clear-cut diagnostic classification, test anxiety is commonly characterized by the fear of evaluation (10), which is associated with various mental and psychophysiological symptoms: Typically, the affected individual suffers from feelings of desperation, mental failure and inferiority, irritability, and depression. Negatively distorted views regarding one’s own abilities and the test situation frequently occur. Other distressing factors are panic-like symptoms. Psychophysiologically, test anxiety is characterized by the activation of the autonomic nervous system, in particular, the activation of the sympathetic and parasympathetic nervous system, resulting in symptoms for example nausea, gastric, intestinal and bladder activation, sweating, increased blood pressure. These physiological symptoms which can be summarized under the psychological term stress, or within classical physiological anxiety symptoms, reflect the high level of mental as well as physical tension. Those symptoms are often correlated with impaired concentration and physical drive, which ultimately make it difficult for the anxious students to complete their studies, or even cope with daily life (11). Also results from the “Health 2000”-study showed the relation between anxiety and depression. It is common, that these two problems occur together (12). Within this scope, Pekrun, Götz, Titz, and Perry (13) showed that excessive anxiety can adversely affect motivation and learning. In addition, individuals suffering from test anxiety display lower test scores than those without test anxiety (14). Furthermore, several studies have shown associations between test anxiety and negative outcome variables, such as diminished academic performance (15) or increased time to graduate (16). Lepp and colleagues found a positive relation between academic performance and life satisfaction. Anxiety, however, was negatively related with satisfaction in life (17). Moreover, another influencing factor is self-efficacy. 14% of the variability in test anxiety can be related to the manifestation of self-efficacy (18). As a result, life satisfaction and self-efficacy of those affected with test anxiety is likely to be reduced. Taking together, it becomes evident that students suffering from test anxiety carry a great psychological as well as physiological burden (4) affecting their academic performance, and possibly as a consequence, their ability to get and practice a job in the competitive work market. Based on these results, it seems important to develop adequate treatment for the disorder.

Thus far, behavioral therapy, cognitive behavioral therapy (CBT) as well as skills training, and biofeedback have proven to be effective in the treatment of test anxiety (19). In addition, relaxation techniques have a long tradition in the treatment of test anxiety (20). Interestingly, although the general incidence for students seeking psychotherapy is increasing, a comparably low number of students is seeking psychotherapy for test anxiety (4, 21). According to Blanko and colleagues (22), less than 20% of students affected by test anxiety in the US receive adequate treatment. Upon evaluating the reasons for not getting treatment for their test anxiety, 25% of the students, who have not yet taken advantage of the service, stated that they were not seeking help due to a lack of time (4). This shows an important problem within students suffering from test anxiety, leading to the need for sufficient short-term interventions for the target group. In order to fill this gap, the present study tailored a new short-term intervention consisting out of two sessions of imagery rescripting (IR) and rehearsal with the aim of reducing students’ test anxiety.

Among several mental disorders, aversive experiences are manifested by intrusive mental images [e.g. (23)]. Imaginations represent perceptual information, arising from memory instead of directly capturing sensory information (24, 25). These mental images can include every sensory modality, for example smell, hearing, or visual content. For those affected, these mental experiences feel just as vivid and real as the actual outside-world occurrences (24). This phenomenon is mostly known within the context of post-traumatic stress disorder (PTSD) as “intrusions”, one of the hallmark diagnostic features of the disorder (26). However, mental images also occur frequently within social phobia, where the individual suffers from negative images of personal failure (27). In a similar way, mental images also occur within individuals experiencing test anxiety (10). These individuals specifically experience thoughts about failure during examination situations, which represent themselves through adverse mental images.

The treatment of adverse mental images has gained increased interest through the development of IR techniques, which are well-established within the context of PTSD and social anxiety (28). Within this technique, mental images are reconstructed and successively modified into benign entities during therapist-guided imagination. As a result, originally negative evaluations and related negative emotions such as anxiety or shame can be modified and connected with more positive experiences of coping with adverse situations (29). A recent meta-analysis reported the effectiveness of IR techniques (30), for several mental disorders like, for example, eating disorders [e.g. (31)], nightmares [e.g. (32)], or personality disorders [e.g. (33)]. The content of the implemented IR techniques typically consisted of the rescripting of past (retrospective) adverse mental images, such as intrusions experienced in PTSD (34), or images of aversive past experiences in social phobia (35). In contrast, until recently, little attention has been given to the occurrence of disturbing prospective mental images related to future events. Within this context, there is evidence for the importance of prospective mental images for bipolar disorders (36), depression, and anxiety disorders (37).

In the context of test anxiety, disturbing mental images of failing in exam situations can be related to retrospective, relived mental image of once experienced situations. Furthermore, the images can be related to imagined exam situations in future with anticipated failure. The application of IR techniques during treatment of test anxiety was first evaluated in a pilot study by Prinz, Bar-Kalifa, Rafaeli, Sened, and Lutz (38) and in a randomized controlled trial (RCT) (39). The program evaluated within the RCT consisted out of five therapy sessions of standard CBT, of which only one was reserved for the application of IR (instead of relaxation techniques). The second group went through the same procedure, with relaxation techniques instead of the IR-session. The third group was a therapist-guided self-help group, which studied and discussed case examples of test anxiety and did not receive any therapeutic intervention (39). Results from the RCT indicate that the combination of CBT and IR was comparably effective as the combination of CBT and relaxation as well as a therapist-guided self-help group. Within the pilot study, six sessions of IR were offered. Subjects included within this study reported a high acceptance of IR techniques, as well as a substantial reduction of anxiety related symptoms at the two-, four-, and six weeks follow-up (Cohen´s *d*= .75; .84) (38).

Based on the above-presented findings, we tailored a short psychotherapeutic intervention with two sessions of IR. Previous findings indicate positive and substantial effects of reducing social anxiety after one session of IR (40), leading to the assumption that comparable effects could be reached within the IR application to test anxiety. In the here-presented two-session IR program, individually relevant distorting mental images associated with the experience of failure during examination were reconstructed and modified in a way that students experience coping with the feared stressful exam event. The aim of the intervention was to reduce negative emotions (fear, shame, sadness, feelings of failure), and replace them with more functional ones. As a result, a reduction of test anxiety should be observable. As in other previous studies (41), we examined the trait component of test anxiety, operationalized by the *Prüfungsangstfragebogen* (PAF), which indicates to measure trait anxiety (42). In addition, the influence on depressive symptoms, life satisfaction, and self-efficacy will be examined based on the research results mentioned above. Since the two-session IR program is the first approach to test anxiety treatment with a pure modular IR application without the embedment in a more comprehensive CBT program, we investigated acceptance and efficacy in a pilot study.

## Material and Methods

### Participants

Nine university students were recruited via flyers and announcements at the local university, social media groups (facebook), e-mail-lists, and the homepage of the University Clinic, Department of Psychiatry and Psychotherapy, Ulm. Interested persons sent their contact information via e-mail and were subsequently contacted for diagnostic telephone screening.

Inclusion criteria were age over 18 years, student status, and self-reported test anxiety before written or oral exams or presentations. In addition, participants were only eligible for participation if they reported disturbing mental images about test situations. Exclusion criteria were current psychotherapy or participation in another (online-) program to reduce test anxiety. In addition, individuals with severe mental disorders (psychotic symptoms, severe depression, etc.) were excluded from the study. Diagnoses were made using the *Mini International Neuropsychiatric Interview* [*M.I.N.I.*, (43)]. Prior to study inclusion, all participants provided written informed consent. All subjects received an indemnity of 40 €.

### Design

This pilot study was implemented as a single intervention with a pre-post design and a 3-month follow-up assessment. Because of the exploratory character of the study, no control group was included. All participants received a two-session IR treatment. The study was conducted under the premises of the University Clinic of Ulm University, Germany, and was approved by the ethics committee of Ulm University, Ulm, Germany.

After the potential subjects expressed their interest in study participation, a short telephone screening was conducted by an experienced clinical psychologist (first author AM). Here, the participants could ask questions, and they went through a short interview. Main inclusion criteria such as self-reported test anxiety and the presence of disturbing test-related mental images were assessed. Telephone screening was followed by a pre-measurement appointment (t1), where subjects underwent a detailed diagnostic procedure by an independent and experienced clinical psychologist, who was not involved in the IR sessions, including a diagnostic interview [*Mini International Neuropsychiatric Interview,* (43)] as well as an adapted version of the *interview of imaginations* (44) in relation to the test anxiety. In addition, participants completed several self-report questionnaires, which are described in more detail at chapter 2.5.2. One week after the pre-assessment (t1) the two-session IR program was administered by a trained clinical psychologist (first author AM) during a two-week period. Post-assessment (t2) was directly conducted after the second IR session, including the processing of various self-assessment tools as well as the evaluation of the intervention by questionnaire. Three months after the post-measurement, a follow-up-survey was conducted in which the subjects also responded to self-assessment questionnaires. Diagnostic assessment at t1, t2, and t3 was applied by an independent clinical psychologist in order to ensure outcome blindness. Adherence to treatment protocol was ensured by weekly applied supervision by the last author (ZSV).

### Intervention

The intervention included two sessions of IR of a test anxiety related mental imagery, which was firstly reconstructed and secondly modified towards a less stressful mental image. Therefore, there was a daily homework of imagery rehearsal between the sessions. The basis for the development of our IR intervention was a published treatment for reducing nightmares (45). It was done an adaption to test-anxiety and a two-session setting.

During the first session, all participating students received psychoeducation about the theory of mental images and their modification through IR. For example, the subjects were introduced to the characteristics of mental images as well as the distinction to verbal thoughts. Since some students reported multiple mental images, the two most vivid or most frequently occurring images were selected for editing in the subsequent sessions of IR. Then the subjects were guided to close their eyes and visualize the discussed mental imagery of the test-situation. All subjects were asked to set out the picture as accurately as possible including as many details of sensory impressions (see, hear, smell, feel), thoughts, emotions, and body reactions as possible. A major focus was paid to the point of no return, which is assumed to be directly followed by a maximum load of test anxiety or blackout. Subsequently, thoughts and feelings, which were closely connected with the test anxiety were defined. Upon completion of the imagery reconstruction, the therapist guided the subjects to convert the mental images by developing another ending. The aim of the modification was the positive management of the situation during imagination, either by the subjects themselves or with the help of a third person. According to typical IR techniques, the new script or story line should not deviate too much from the original imagination. A large associative proximity to the original script was of great importance (45). Imagery modification was written down and recorded for the sake of imagery rehearsal during daily homework. It was ensured that the text included the wording of the subjects. Furthermore, the subjects were asked to choose whether themselves or the therapist should record the modified imagery at audio tape or mobile phone. The subjects were encouraged to practice the new, comfortable mental image. Hence, participants were instructed to listen to the audio recording of the rescripted imagination at home once a day. In order to control how reliably this task was performed, the subjects filled out a daily protocol, stating how often they had practiced the imagination and how vivid the imagery was. In addition, they provided information on their well-being on a second record sheet, as described in more detail in section 2.5.3. During the second session, the daily rehearsal protocols were collected and discussed, especially how often the students could implement the homework or whether further modifications were necessary for treating a second mental imagery of test anxiety. Moreover, it was asked for any deterioration caused by the procedure (for example sleeping disturbance, changes in mood). Afterwards, the already discussed second mental image, which was usually less disturbing than the first, was modified. The procedure was the same as to the rescripting during session one. As the conclusion of the session, the subjects could ask questions and give feedback. The therapist encouraged the participants to continue the imagery exercises and carry out IR for in case of future emerging, stressful imaginations.

### Measures

#### Diagnostic Interviews

To assess possible psychiatric disorders, the *Mini International Neuropsychiatric Interview* [*M.I.N.I.*, (43)] was carried out. Test-anxiety related mental imagery was assessed via interview which was a combined adaption of the semi-structured interview for mental imagery (44) and the “Intrusion Interview” by Patel and colleagues (46).

#### Self-Ratings

The following variables were recorded using different questionnaires: Socio-demographic information like gender, age, nationality and marital status, as well as the use of health care services with respect to test anxiety (*Socio-demographic questionnaire),* test anxiety [*Prüfungsangstfragebogen PAF* (42)], depressive symptoms [*Beck Depression Inventory-II BDI* (47)], life satisfaction [*Fragebogen zur Lebenszufriedenheit FLZ* (48)], self-efficacy [*Fragebogen* *zur allgemeinen Selbstwirksamkeit WIRKALL_r* (49)], study-specific self-efficacy [*Fragebogen zur studienspezifischen Selbstwirksamkeit WIRK_STUD* (50)], psychometric assessement of change and acceptance [*Veränderungsfragebogen des Erlebens und Verhaltens VEV* (51); *Bonner Fragebogen für Therapie und Beratung BFTB* (52)], patient satisfaction [*Fragebogen zur Messung der Patientenzufriedenheit ZUF* (53) and mental imagery (*Vividness of Visual Imagery Questionnaire VVIQ* (54); *Impact of Future Events Scale IFES* (55); *Spontaneous Use of Imagery Scale SUIS* (56)]. For assessment of academic success, two items were added: “How do you rate your own academic achievements/your academic success?” as well as “Please enter your current average grade (take into account the achievements of the last examination period).”

#### Protocols Between the IR-Sessions

Daily protocols between the two IR sessions were filled out by all participants.

*Implementation and vividness of the imagination*. The first protocol recorded the frequency of performing the IR homework and the vitality of the imagery. Here, the subjects ranked how vividly they could see/hear/smell/taste/feel something on a scale of 0 to 100.

*Side effects and control variables*. The second protocol was used to record possible current stressors that could influence the effect of the intervention. Furthermore, possible side effects were controlled. On a scale from 0 to 10, all participants rated their mood, the degree of test anxiety and problem behavior that might have occurred (for example sleeping disturbance, chewing fingernails, smoking, etc.).

### Statistical Analysis

The data was analyzed using SPSS Statistics Version 25 (57) for Microsoft Windows. No cases were excluded from the analysis.

To test the overall changes, an ANOVA for repeated measures was used. If requirements for this procedure were not fulfilled, a Friedman Test was calculated. Post-hoc contrasts were calculated. In addition, descriptive analyses of satisfaction and acceptance were performed (absolute frequencies, mean values, medians, standard deviations). Effect size was calculated as Cohen´s *d* (58). For this purpose, the online-based effect size calculator from Psychometrica was used. The values can be interpreted as follows: .20 weak, .50 medium, .80 large effect (58). The alpha error was set at α=.05. Due to the exploratory character of the study, correction for multiple testing was not applied and *p*-values up to .1 were interpreted as trends.

## Results

### Sample Characteristics

All nine participants (66.7% female) were Caucasians aged 21 to 31 years (*M* = 23.11 years; *SD* = 3.22). 33.3% were in a relationship. All subjects were German native speakers. The majority (88.9%) had never been in psychotherapeutic treatment. All subjects had an upcoming exam: 66.7% of the subjects had an upcoming exam in the next four weeks after pre-measurement, two of them (22.2%) within the following two weeks, and 33.3% within 9 weeks or later. About half of the sample (44.4%) was in exam preparation. 66.7% rated their own previous performance in the study as average, 22.2% as above average and 11.1% as below average. The mean grade of the sample was *M* = 2.04 (*SD* = .40), with 1 reflecting the best grade. In addition to test anxiety, the sample included other mental disorders at t(1) major depression (moderate episode) (11.1%), social phobia (44.4%), generalized anxiety disorder (11.1%).

### Study Flow

**Figure 1:**
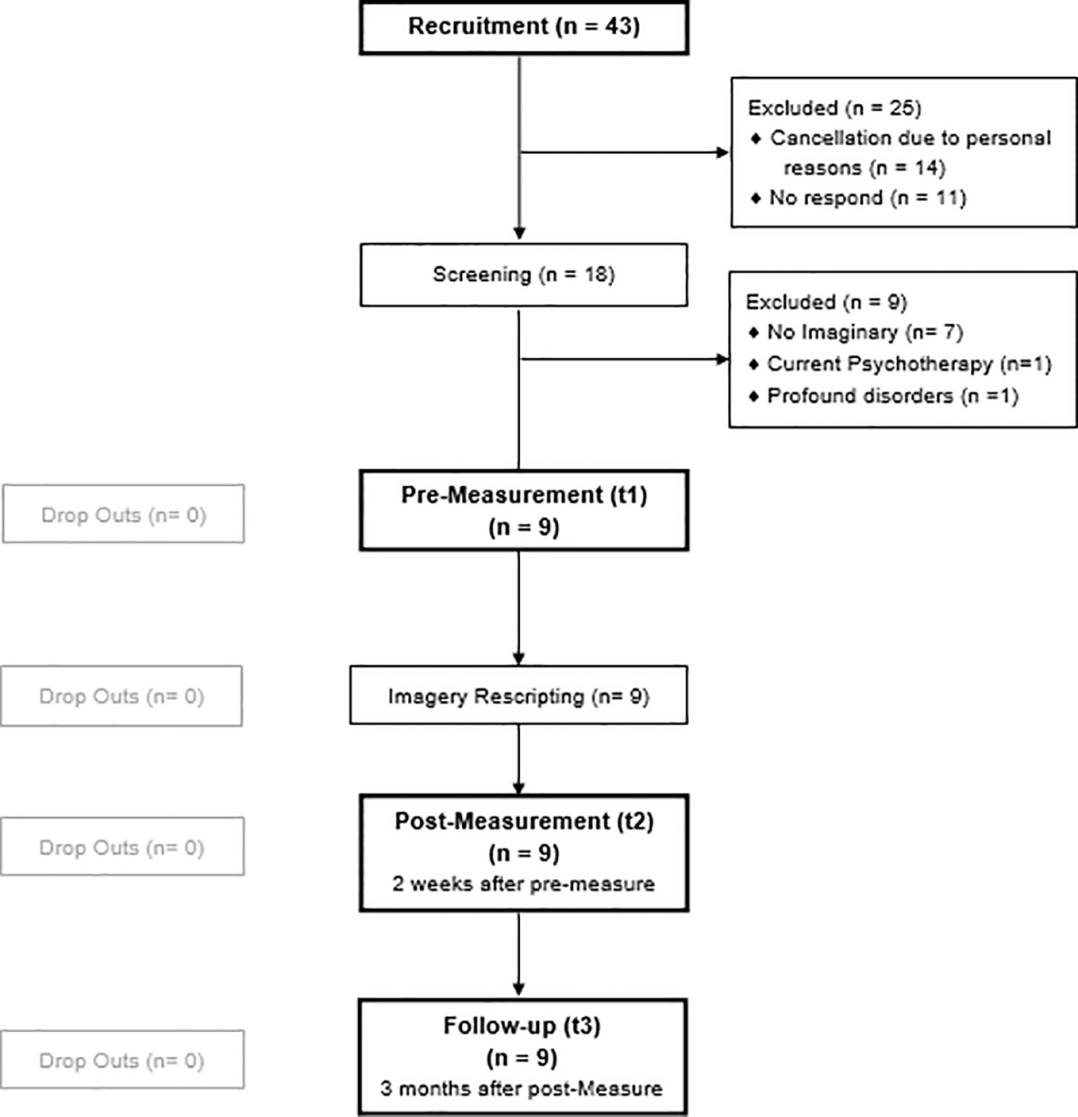
Participants flow chart.

In total, 43 individuals replied to our study announcement. Among these, 18 registered for the telephone screening. After application of exclusion criteria, nine subjects remained for study inclusion. Seven of the exclusions were due to lack of test-anxiety related mental imagery, one participant suffered from autism, and one was in a current psychotherapy. All nine participants, which finally enrolled in the study, participated in all measurements (at pre-, post-, and follow-up measurement). Dropouts and study flow are shown in **Figure 1**.

### Previous Use of Health Care Services

One participant has previously received psychotherapy with respect to past test anxiety. No participant consulted a psychiatrist. Experiences with other health care services were more common: 33.3% turned to a counseling center, 11.1% used help services via the internet.

### Treatment Acceptance and Satisfaction

All subjects completed the study including the 3-months follow-up measurement. The dropout rate after intervention was 0%, as illustrated in the flowchart (**Figure 1**). All participants stated that they did the homework on a regular basis, on average on 5.1 days a week (*SD* = 2.10, *range* = 0-7) between pre- and post-measurement. From t(2) to follow-up, most subjects (66.7%) exercised the imagery rehearsal once a week, one subject (11.1%) practiced rehearsal only once per month and two subjects (22.2%) even rarer.

#### Process Scale of BFTB

Beside the measurement of specific outcome effects due to the application of IR, unspecific therapy effects related to therapist behavior were additionally assessed by the Bonner Fragebogen für Therapie und Beratung (*BFTB, Questionnaire for Therapy and Consultation*). Hence, the BFTB was obtained only after completion of the intervention at t2. The analysis of the BFTB process scale showed the following descriptive statistics, displayed in **Table 1**.

**Table 1:** Therapeutic process according to BFTB: Means and standard deviations, range, possible range and t-values at t2 (*n* = 9).

	*M(SD)*	Range	Possible Range	t-value
Empathy	42.00 (4.82)	33-50	10-50	50
Authenticity	46.00 (2.92)	42-50	10-50	54
Appreciation	43.00 (1.94)	39-42	10-50	44
Interpretation	25.11 (7.22)	10-33	10-50	37
Consciential	27.88 (4.16)	22-34	8-40	48
Structuring	47.56 (3.05)	42-50	10-50	61
Confrontation	26.44 (6.51)	15-37	10-50	45
Work trough	25.00 (6.71)	12-34	10-50	38
Emotion-centered work	44.89 (5.56)	36-51	11-55	51
Reinforcement	42.22 (3.50)	36-49	10-50	50

#### ZUF-8

The participants´ satisfaction with the intervention ranged from 24 to 32 (*M* = 27.78, *SD* = 2.86) on a possible scale from 8 to 32. The median was 27.00.

### Efficacy

Descriptive statistics of the outcome variables (*PAF, BDI, WIRKALL, WIRKSTUD*, and *IFES*) are shown for all participants before treatment (t1), after treatment (t2), and at the 3-month follow-up in **Table 2**.

**Table 2:** Changes in outcome variables over time: Means, standard deviations, test statistics and effect sizes at baseline (t1), post-treatment (t2), 3 months follow-up (t3) (*n* = 9).

	t1 *M (SD)*	t2 *M (SD)*	t3 *M (SD)*	Test statistic	Effect size *^a^* t1 – t2	Effect size *^a^* t1 – t3
*PAF*	30.78 (12.26)	31.00 (13.37)	23.67 (8.08)	*F*(2,16)=4.11, *p*=.036 ^b^	.037	.715
*BDI II*	8.89 (6.79)	7.22 (5.47)	7.00 (5.66)	*χ2* (2) = 2.69, *p*=.261 ^c^	.446	.267
*FLZ*	257.38 (40.51)	252.25 (44.16)	266.50 (36.96)	*F* (2,14)=1,60, *p*=.237 ^b^	.156	.309
*WIRKALL*	27.67 (5.22)	28.11 (5.56)	30.67 (4.06)	*F* (2,16)=12.46, *p*=.020 ^b^	.167	1.16
*WIRKSTUD*	17.56 (3.40)	18.11 (3.18)	19.78 (2.77)	*χ2 (*2) = 12.80, *p*=.002 ^c^	.295	1.49
*IFES*	31.22 (14.31)	19.56 (10.37)	21.63 (9.83)	*F (2,14)=25.20, p*=.000 ^b^	.543	.926
*Average grade*	2.04 (0.40)	2.01 (0.34)	1.99 (0.56)	*χ2* (2) = 1.04, *p*=.595 ^c^	.465	.135

Test anxiety questionnaire “PAF, Prüfungsangstfragebogen”; “BDI-II, Becks Depression Inventory”; Life satisfaction questionnaire “FLZ, Fragebogen zur Lebenszufriedenheit”; General self-efficacy questionnaire “WIRKALL, Fragebogen zur allgemeinen Selbstwirksamkeit”; Study-specific self-efficacy questionnaire “WIRKSTUD, Fragebogen zur studiumsspezifischen Selbstwirksamkeit” “IFES, Impact of Future Event Scale”Average grade.

^a^Cohen’s d for repeated measures, ^b^ANOVA with repeated measures, ^c^Friedman Test.

**Table 2** also shows changes with respect to academic grades over time [*χ2* (2) = 1.04, *p* = .595]. There was a small, however not significant difference across the measurement times. A similar trend emerged with respect to the self-assessment of the study performance. While at t1 and t2, 22.2% classified their study performance as above, 11.1% as below, and 66.7% as average, at t3 none of the participants reported below average performance. In contrast, 77.8% classified their study performance at t3 as average and 22.2% above average.

#### Test Anxiety

**Figure 2:**
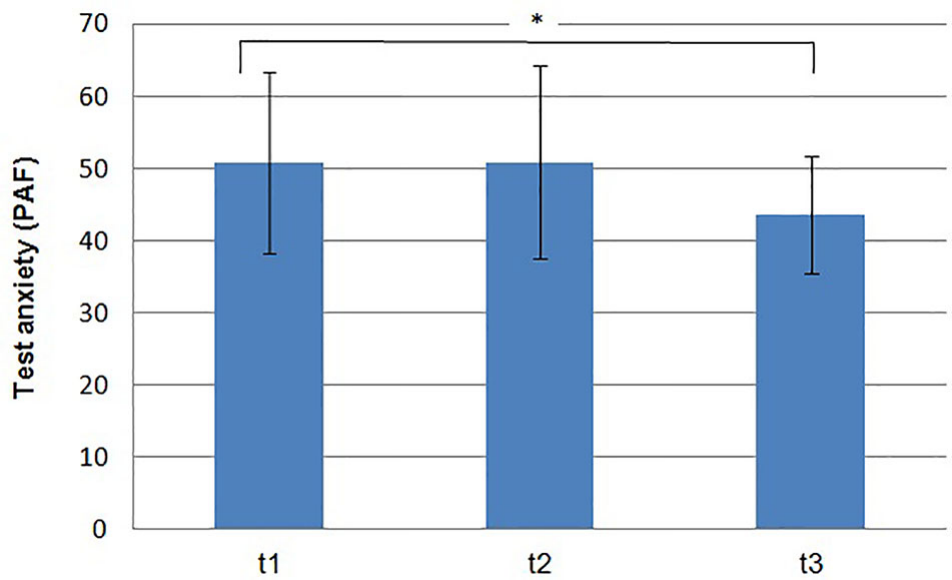
Means and standard deviations of the test anxiety on pre- *(t1)*, post measurement *(t2)* and follow up *(t3)*. (*p < .05).

Test anxiety significantly dropped over time [*F*(2,16) = 4.11, *p *= .036, partial *η²* = .339]. The contrasts showed a significant improvement from pre-measurement (*M *= 30.78, *SD *= 12.26) to follow-up (*M *= 23.67, *SD *= 8.08) of 7.11 (*SE *= 3.03, *p *= .047), whereas a trend from post-measurement (*M *= 31.00, *SD *= 13.37) to follow-up (*M *= 23.67, *SD *= 8.08) of 7.33 (*SE *= 3.44, *p = .*065) could be observed (see **Figure 2**). The effect size from t1 to t3 was *d* = .715.

#### Depressive Symptoms

A Friedman Test was conducted in order to investigate the change over time in depressive symptoms as assessed with the *BDI-II*. There was no significant change and/or exacerbation in depressive symptoms over time [*χ2* (2) = 2.69, *p* = .261].

#### Life Satisfaction

Due to missing values, the subscales “relationship to the own children”, “marriage and partnership”, and “work” were excluded from the analysis of life satisfaction, measured via *FLZ*. The ANOVA, based on the remaining subscales, revealed no differences in life satisfaction between the three measurement point [*F* (2,14) = 1.60, *p *= .237, partial *η²* = .186].

#### General Self-Efficacy

The general self-efficacy, measured by *WIRKALL*, showed an significant change over time [*F* (2,16) = 12.46, *p *= .020, partial *η²* = .387]. The analysis of contrasts revealed a significant improvement of general self-efficacy from pre-measurement (*M* = 27.67, *SD* = 5.22) to follow-up (*M* = 30.67, *SD* = 4.06) of 3 (*SE* = .85, *p* = .008). There was a trend from post-measurement (*M* = 28.11, *SD* = 5.56) to follow-up (*M = *30.67, *SD* = 4.06) of 2.56 (*SE* = 1.25, *p* = .075).

#### Study Related Self-Efficacy

Friedman Test revealed that study related self-efficacy as assessed by *WIRKSTUD* increased significantly over time [*χ2 (*2) = 12.80, *p* = .002]. Post hoc tests revealed a significant difference between pre-measurement and follow-up (*z* = -2.828, *p* = .005). Also, there was a significant change from post-measurement to follow-up (*z* = -2828, *p* = .005).

#### Mental Imagery

The personal distress caused by intrusive prospective mental imageries related to test anxiety as measured by the *IFES* changed significantly over time *[F (2,14) = 25.20, p* = .000, partial *η²* = .783]. There was a significant reduction between pre-measurement (*M* = 37.13, *SD* = 9.66) and post-measurement (*M* = 18.13, *SD* = 10.23) of 19 (*SE* = 3.39, *p* = .001) and also from pre-measurement (*M* = 37.13, *SD* = 9.66) to follow-up (*M* = 22.25, *SD* = 10.46) of 14.88 (*SE* = 2.64, *p* = .001).

## Discussion

The present study implemented a short-term intervention consisting out of two stand-alone IR sessions for individuals suffering from test anxiety. Our exploratory investigation examined the acceptance and efficacy of this new approach, implemented to reduce test anxiety.

We found a high acceptance of the implemented two-session IR intervention: There were no dropouts. All included subjects completed the treatment sessions, as well as the diagnostic appointments at various time-points. This finding is in line with other studies investigating IR in general, and test anxiety in particular. Within these studies, no or only low dropout rates (38, 59) were reported, suggesting high levels of acceptance for IR techniques. In addition, there was a high adherence to carry out the homework regularly. Also, the majority of participants continued the imagination of the newly created mental image for at least three months until the follow-up. Furthermore, our analysis revealed high satisfaction with the therapy, as well as with the therapist, reflected by good overall BFTB scores. This result additionally indicates acceptance of the participants with the intervention. Subjects positively rated aspects such as session structuring and emotion-focus. All participants perceived the intervention as helpful and of good quality, as reflected by the results of the ZUF-8. In conclusion, as previous data have shown that test anxiety affected individuals are more likely to use low-threshold services such as online interventions or counseling centers than to attend long-term treatments such as consultation of a psychiatrist or psychotherapist, our present findings could indicate a stronger preference towards short-term interventions within this group, and might reflect the need of more short-term interventions in the treatment of such anxiety. Such an intervention could also be offered by psychologists and psychotherapists through workshops or courses in schools and universities, without the need for psychotherapy. This might further reduce the threshold for usage.

We evaluated efficacy of our applied short-term intervention based on self-reports as assessed by PAF and BDI-II. Both diagnostic instruments indicated that the symptoms did not increase. Overall, we were able to observe a symptom reduction for test anxiety over time. The significant decrease from t(1) to the three-months follow-up revealed a medium to large effect according to Cohen’s d. This is consistent with the results of a meta-analysis on IR [(30) *g* = 1.22] as well as with studies on IR in test anxiety, which reported equivalent large effect sizes [(39): *d* = 1.66; (38): *d* = .84]. Descriptive changes in other emotions continued to appear. Unpleasant feelings such as hopelessness and anger decreased or remained the same, while positive emotions such as lust, joy, and relief increased. The latter two increases were significant (p <. 05). Furthermore, there was an improvement of general self-efficacy and study-related self-efficacy. Our observed large effect sizes (*PAF*: *d* = .715, *WIRKALL_r*: *d* = 1.16, *WIRK_STUD*: *d* = 1.49) indicate a high degree of clinical significance. Finally, we were able to confirm that the influence, and thus the burden of mental images associated with test anxiety decrease over time. The overall IFES score declined substantially after treatment application and was associated with a large effect size (*d* = .926). Thus, as a possible result of our IR training, students reported less frequent and less intrusive mental images with respect to exam situations. Further support for the potential efficacy of the intervention was found with respect to the observed trend for improvement of academic grades and study performance. However, these differences was not substantial, which again may have been due to the small sample size. In addition, academic grades develop over an entire school or university year, making it possible than our here investigated time window of 3 months might have been too short in order to adequately identify substantial grade changes. Further studies implementing longer follow-up periods could be helpful for future studies. However, our preliminary results might give initial indication of the effectiveness of the procedure.

Several factors might explain the reduction in test anxiety. On the one hand, mental imagery uses the same neural mechanisms as perception of a real situation (25, 60). As a result, the students may have been able to develop real-life coping strategies during the imagery. Imaging a successful exam might have strengthened neural pathways of experiencing real success, which in turn might have increased the subjective evaluation of being able to handle exams in terms of self-efficacy. This assumption is consistent with our finding that IR has not only reduced test anxiety, but also boosted participants’ self-efficacy. Social cognitive theory by Bandura [(61) p. 77] has described the "central role of perceived self-efficacy to exercise control over potential threats in anxiety arousal”. Within the context of test anxiety, several findings indicate a negative correlation between self-efficacy and the level of test anxiety. Accordingly, individuals experiencing lower self-efficacy expect stronger test anxiety (18). Another possible explanation could be habituation. Habituation as a result of frequent confrontation with fear or the fear-eliciting situations is considered substantial in the therapeutic reduction of fears. In the present study, habituation could have occurred as a result of daily rehearsal of the more benign mental image.

Our results in terms of acceptance and effectiveness of the intervention are consistent with two recently published pioneer studies on this topic (38, 39). However, the present study is the first to implement IR as a stand-alone technique, which taken together with previous studies provides some initial, but solid evidence for its effectiveness in the course of treating test anxiety with no serious side effects to be expected.

### Limitations

Despite the promising preliminary results, the previous study is of an exploratory nature, and results must be interpreted with caution due to several limitations. First, the present study is limited by the sample size. This increases the likelihood of underpowering, and of incidental findings (62). Accordingly, even though our reported effect sizes are moderate to high, the small sample size limits interpretation of the results and statements about clinical significance. The generalizability of the data is limited. Second, there was no control group. Again, this reduces the generalizability of the results since factors such as maturation effects or reactivity are not sufficiently controlled. Therefore, treatment response must be interpreted with caution. Third, in our study only students from universities and colleges were included. It remains questionable whether the results can also be transferred to pupils with test anxiety, vocational students, or other groups. Furthermore, in our sample women were predominantly represented, which reflects the gender-specific appearance of test anxiety (63). In consequence the results cannot simply be generalized to male test anxiety sufferers. Fourth, there is the possibility of a selection bias, since predominantly motivated people were willing to take the time and effort to participate in the study. Fifth, long-term effects of the intervention cannot be reliably derived, as the period of three months from t1 to follow up was very tight. In summary, further confirmatory studies are necessary.

### Conclusion

This is the third study which examined IR in test anxiety. At the same time, it is also the first study that offers IR as an isolated intervention and does not embed it into another treatment that has already proven to be effective. Therefore, despite the exploratory character and the associated limitations, which demand careful interpretation, the present study provides important information that could stimulate future research. The intervention with only two sessions and no additional therapy seems acceptable and helpful for individuals suffering from test anxiety.

## Data Availability Statement

The datasets generated for this study are available on request to the corresponding author.

## Ethics Statement

The studies involving human participants were reviewed and approved by Ethical Board of University of Ulm. The patients/participants provided their written informed consent to participate in this study.

## Author Contributions

AM: Study conception and design, analysis and interpretation of data, manuscript writing and editing. CS: Study design, manuscript editing. JK: Study design, manuscript editing. BC: Study design, manuscript editing. ZS-V: Study conception and design, interpretation of data, manuscript editing, critical revision.

## Conflict of Interest

The authors declare that the research was conducted in the absence of any commercial or financial relationships that could be construed as a potential conflict of interest.
